# Bacteria in the Air

**Published:** 1881-05

**Authors:** 


### BACTERIA IN THE AIR.

According to P. Miquel, the germs of bacteria are almost always in the air, but their quantity undergoes great variation. They are very few in winter, increase in spring, remain abundant in summer and fall, and then diminish rapidly with the beginning of frost. In this regard they are like the spores of mould parasites, but while the mould spores increase in moist weather, the bacteria diminish.

In Montsouris are sometimes found in summer and fall 1,000 germs of bacteria in one cubic inch of air, in winter they reduced four or five times and then it sometimes happens that it is necessary to pass 200 liters of air through a solution of sterilized meat extract to induce in it the development of bacteria. In the interior of houses thirty to fifty liters suffice. But in his laboratory the author required only five liters to secure infection. From observations which the author made in Paris from December, 1879 to June, 1880, it was seen that *every increase in the air bacteria, was followed in the course of eight days, by an increase in the number of deaths from contagious and epidemic diseases.* The author further observed that the air over decomposing matter is free of air bacteria. Concering which he has more to report at a later date. —*Compt. rend.*, xci., No. 64.—J. T. W.

